# RU-Net: skull stripping in rat brain MR images after ischemic stroke with rat U-Net

**DOI:** 10.1186/s12880-023-00994-8

**Published:** 2023-03-27

**Authors:** Herng-Hua Chang, Shin-Joe Yeh, Ming-Chang Chiang, Sung-Tsang Hsieh

**Affiliations:** 1 Computational Biomedical Engineering Laboratory (CBEL), Department of Engineering Science and Ocean Engineering, National Taiwan University, No. 1 Sec. 4 Roosevelt Road, Daan, Taipei 10617 Taiwan; 2 Department of Neurology and Stroke Center, National Taiwan University Hospital, Taipei, 10002 Taiwan; 3 Department of Biomedical Engineering, National Yang Ming Chiao Tung University, Taipei, 11221 Taiwan; 4 Graduate Institute of Anatomy and Cell Biology, College of Medicine, National Taiwan University, Taipei, 10051 Taiwan; 5 Graduate Institute of Clinical Medicine, College of Medicine, National Taiwan University, Taipei, 10051 Taiwan; 6 Graduate Institute of Brain and Mind Sciences, College of Medicine, National Taiwan University, Taipei, 10051 Taiwan; 7 Center of Precision Medicine, College of Medicine, National Taiwan University, Taipei, 10051 Taiwan

**Keywords:** Skull stripping, Brain segmentation, Deep learning, U-Net, Ischemic stroke, MRI

## Abstract

**Background:**

Experimental ischemic stroke models play a fundamental role in interpreting the mechanism of cerebral ischemia and appraising the development of pathological extent. An accurate and automatic skull stripping tool for rat brain image volumes with magnetic resonance imaging (MRI) are crucial in experimental stroke analysis. Due to the deficiency of reliable rat brain segmentation methods and motivated by the demand for preclinical studies, this paper develops a new skull stripping algorithm to extract the rat brain region in MR images after stroke, which is named Rat U-Net (RU-Net).

**Methods:**

Based on a U-shape like deep learning architecture, the proposed framework integrates batch normalization with the residual network to achieve efficient end-to-end segmentation. A pooling index transmission mechanism between the encoder and decoder is exploited to reinforce the spatial correlation. Two different modalities of diffusion-weighted imaging (DWI) and T2-weighted MRI (T2WI) corresponding to two in-house datasets with each consisting of 55 subjects were employed to evaluate the performance of the proposed RU-Net.

**Results:**

Extensive experiments indicated great segmentation accuracy across diversified rat brain MR images. It was suggested that our rat skull stripping network outperformed several state-of-the-art methods and achieved the highest average Dice scores of 98.04% (p < 0.001) and 97.67% (p < 0.001) in the DWI and T2WI image datasets, respectively.

**Conclusion:**

The proposed RU-Net is believed to be potential for advancing preclinical stroke investigation and providing an efficient tool for pathological rat brain image extraction, where accurate segmentation of the rat brain region is fundamental.

## Background

Stroke is the leading cause of serious long-term disability and the major cause of mortality worldwide [1]. Of all strokes, the majority are the ischemic type resulting from the occlusion of a cerebral artery by a blood clot. Cerebral ischemia can induce many injuries including energy failure, intracellular calcium overload, and cell death, which eventually lead to the loss of neurological functions and permanent disabilities [2]. Experimental ischemic stroke models are crucial to understand the mechanism of cerebral ischemia and to evaluate the development of the pathological extent. Among the models in a variety of species, rodent stroke models have been broadly employed in experimental ischemia studies for decades [3].

In particular, the transient middle cerebral artery occlusion (tMCAO) model in rats is one of the closest simulations of human ischemic strokes, which has been frequently utilized to induce infarction at the basal ganglion and cerebral cortex [4, 5]. To noninvasively disclose stroke regions and the associated tissue, one popular manner is through the use of magnetic resonance imaging (MRI), where diffusion-weighted imaging (DWI) and T2-weighted MRI (T2WI) exhibits complementary visualization of ischemic lesions [4]. A fundamental task of the preclinical MRI studies associated with tMCAO models is the skull stripping in rat brain MR images. Skull stripping, also known as brain extraction or intracranial segmentation, is a process to remove nonbrain tissues and separate brain regions in MR images. The extracted rat brain is critical to succeeding processes such as hemisphere segmentation, lesion segmentation, tissue classification, and volume measurement in preclinical stroke investigation [6–8].

Unfortunately, computer-aided tools for rat brain extraction have been lacking. Manual delineation of the rat brain region on numerous MR images has been widely adopted in many preclinical studies [3, 8, 9], which is a time-consuming and laborious work with low reproducibility [10, 11]. In consequence, an accurate and reliable image segmentation tool for the brain extraction in MR image volumes is essential in experimental stroke rat analysis. Automatic skull stripping in rat brain MR images is quite challenging as typical magnetic fields are higher ($\ge 7\text{T}$ commonly) with a larger degree of radiofrequency inhomogeneity, which results in susceptibility artefacts and field biases [12]. Nevertheless, several attempts have been made to address the brain extraction problems in rat MR image volumes. For example, Li et al. [13] presented an automatic rat brain extraction method called the rat brain deformation (RBD) model, which made use of the information on the brain geometry and the T2WI image characteristics of the rat brain.

A fully automatic skull stripping method in an atlas-based manner was proposed for rat MRI scans [14], which was founded on an iterative, continuous joint registration algorithm. Lancelot et al. [15] developed a multi-atlas based method for automated anatomical rat brain MRI segmentation in such a way that MR images are registered to a common space, where a rat brain template and a maximum probability atlas were constructed. Delora et al. [16] presented a template-based brain extraction scheme called “SkullStrip” to segment the whole mouse brain in T1-weighted and T2-weighted MR images. Huang et al. [17] built a statistic template of the rodent brain, which was adopted to predict the location of the brain in MR images. Alternatively, Zhang et al. [18] combined deformable models and hierarchical shape priors, which constrain the intermediate result for rodent brain structure segmentation. Oguz et al. [19] introduced a rapid automatic tissue segmentation (RATS) algorithm based on grayscale morphology with initial surface extraction followed by graph search. Liu et al. [10] described an automatic brain extraction method, entitled SHape descriptor selected Extremal Regions after Morphologically filtering (SHERM), which extracted the brain tissue in both rat and mouse MR images.

With recent advances in artificial neural networks, many researchers have demonstrated their effectiveness in human brain image segmentation [20–22]. However, few studies have applied this strategy for rodent brain extraction comparing to human brain investigation [23]. The major difference between the human and rodent brain extraction results from the inherent brain dissimilarity in many aspects including the brain tissue geometry, brain-scalp distance ratio, tissue contrast around the skull, partial volume effect with respect to image resolution, and more noise due to a stronger magnetic field in rat brain MRI. One example is the automatic cropping scheme based on the pulse coupled neural network (PCNN) with a slice-by-slice fashion, which was proposed to segment the rat brain in T2WI image volumes [24]. Afterward, Chou et al. [25] described an automatic rodent brain extraction method by extending the PCNN algorithm into 3-D, which operated on the entire rodent brain MR image volume. Recently, deep learning-based approaches have shimmered the field of computer vision in that convolutional neural networks (CNNs) have been successfully applied in many image processing tasks, e.g., classification of the ImageNet database [26]. To handle semantic segmentation problems, the fully convolutional network (FCN) [27], which is an end-to-end and pixel-to-pixel network, has shown its outstanding performance over the CNN. In contrast to the CNN models, the FCN framework exploits an upsampling tactic instead of the fully-connected layer to recover the intermediate image back to the original image dimension.

One particular type of FCN architectures, U-Net [28], has been shown valuable in biomedical image segmentation and it has become the foundation of many segmentation methods. For example, an end-to-end learning algorithm for medical image segmentation was proposed [29], which introduced a category attention boosting module into the 3D U-Net segmentation network. A stacked U-Net scheme was applied to computed tomography image reconstruction that generated high-quality images in a short time with a small number of projections [30]. An automatic hemorrhagic stroke lesion segmentation approach in computed tomography scans was described, which is based on a 3D U-Net architecture incorporating the squeeze-and-excitation blocks [31]. For preclinical studies, Hsu et al. [32] employed the U-Net to automatically identify the rodent brain boundaries in MR images, which was trained and evaluated using rat and mouse datasets. De Feo et al. [33] presented a multi-task U-Net (MU-Net) framework that was designed to accomplish both skull stripping and region segmentation in large mouse brain MRI datasets. In light of the U-Net architecture, the final block of the decoder branch bifurcates into two different output maps corresponding to the two tasks. A unique CNN, called MedicDeepLabv3+ [34], was introduced to simultaneously segment intracranial brains and cerebral hemispheres in rat brain MR image volumes. By incorporating spatial attention layers and additional skip connections into the decoder, the network was able to attain more precise segmentation.

Stimulated by the demand of the preclinical ischemia studies, this paper develops an automatic skull stripping framework in rat brain MR images after stroke based on a deep learning network. The proposed architecture takes advantage of U-Net [28], residual network [35], and batch normalization [36] to perform efficient end-to-end segmentation in rat brain images, which is named Rat U-Net (RU-Net) and publicly available at https://github.com/lvanna/RU-Net. With the same U-shape like structure, two different skull stripping networks are individually trained and validated using two different MRI modalities of DWI and T2WI images. Due to the deficiency of public rat brain MR images after ischemic stroke, two in-house datasets corresponding to DWI and T2WI have been established. Skull stripping in the two MRI modalities using the proposed RU-Net is fairly compared with the state-of-the-art methods. The main contributions of the current work are summarized as follows:

1. A new skull stripping system, referred to as RU-Net, specifically designed for handling pathological rat brain MR images after stroke was developed.
2. On the foundation of a U-shape like architecture, a batch normalization associated with residual network strategy was investigated for extracting the rat brain characteristics.
3. A pooling index transmission mechanism between the encoder and decoder was introduced to tackle large intensity variations in ischemic rat brain MR images.
4. Two in-house datasets containing pathological rat brain DWI and T2WI image volumes were established.

The remainder of this paper is organized as follows. In Sect. 2, we describe the acquired datasets followed by the deep learning architecture for effective feature extraction and elaborate the proposed RU-Net for rat skull stripping. Section 3 presents experimental results and performance analyses regarding both modalities of DWI and T2WI image data. Section 4 discusses our investigation pertaining to the segmentation outcome. Finally, we draw the conclusion in Sect. 5.

## Materials and methods

### Ischemic stroke model

An ischemia-reperfusion model of rats based on the tMCAO with a silicon-coated nylon filament was carried out. Supplied by BioLASCO Taiwan Co., male *Sprague-Dawley* rats with ages of 7–9 weeks old and body weights of 181–336 g were employed as experimental subjects. Different ischemic durations of 0.5, 0.75, 1, 1.5, 2, and 3 h were performed to develop a wide range of infarction. Before the operation, the rats were kept under standard conditions and supplied with water and food ad lib. Under inhalation anesthesia with isoflurane (induction dosage: 4%, maintenance dosage: 2%), anterior neck incision at the right paramedian line (5 mm from the midline) was executed to disclose the right carotid artery. After serial ligations of the right common carotid artery (CCA), external carotid artery, and internal carotid artery (ICA), a silicon-coated filament was inserted into the right CCA and deliberately advanced towards the right ICA until a light resistance encountered. The filament sizes were determined in accordance with the body weight of each individual rat. The rats were allowed to regain consciousness after fixation of the filament on ICA followed by closure of the neck wound. Toward the end of the ischemic period, the rats received anesthesia again for removing the filament to accomplish reperfusion. In accordance with the principles of the Basel Declaration, the protocol was approved by the Animal Committee of National Taiwan University College of Medicine.

### Image acquisition

This study was dedicated to the skull stripping of pathological rat brain MR images with cerebral ischemia. Since there is no public image dataset that is appropriate for our investigation, we have established two in-house preclinical stroke rat MRI datasets. Each stroke rat experienced DWI and T2WI examination for unveiling ischemic regions in the brain. All rat MR images were acquired using the 7T MRI machine (Bruker PharmaScan, Ettlingen, Germany) at National Taiwan University, Taipei, Taiwan. The parameters of the DWI sequence were as follows [37]: b-value 1000 s/mm^2^, repetition time (TR) 4500 ms, echo time (TE) 30 ms, coronal section thickness 1 mm with 15 slices, field of view (FOV) $2.56\times 2.56$ cm^2^, and matrix size $128\times 128$. The parameters of T2WI were as follows: 15 contiguous, coronal slices (thickness: 1 mm) acquired with an FOV of $2.56\times 2.56$ cm^2^, matrix size $256\times 256$, TR 3000 ms, and TE 50 ms. Altogether, there were 55 rat subjects captured with DWI and T2WI for this study. After the MRI scanning, the rats were sacrificed for in vitro staining experiments. All rats were euthanized by intracardiac infusion of 1% sodium nitrite under inhalation anesthesia of isoflurane at 5% through a vaporizer in a dedicated euthanasia chamber.

### Data preprocessing

To generalize the proposed algorithm when handling heterogeneous image data, a least possible preprocessing step was first executed. Specifically, the standard score (or z-score) normalization [38] was exploited to reduce the intensity variation while maintaining the detailed structures of the input rat brain MR images. The standard score is the signed fractional number of standard deviations that is frequently utilized to standardize scores on the same scale by dividing a score’s deviation in a dataset. Mathematically, the input rat brain MR image scan $I$ is normalized with

1 $$\widehat{I}(x,y)=\frac{I(x,y)-{\mu }_{I}}{{\sigma }_{I}}$$

where ${\mu }_{I}$ is the mean intensity of the images in the dataset, ${\sigma }_{I}$ is the corresponding standard deviation in the image dataset, and $\widehat{I}$ is the standardized rat brain MR image.

An essential role to deep learning-based investigation is the use of tremendous image data in the model training phase. For biological image processing applications as in our scenario, the number and scope of images are substantially limited comparing to many famous image databases such as ImageNet. In consequence, data augmentation, which is a strategy to expand the amount of data by generating modified copies or newly created images from existing data, has been commonly adopted as a regularizer to lessen overfitting [26, 39]. To increase the scale and diversity of the acquired rat brain MR image data, we employed four distinct forms of data augmentation, which allowed transformed images to be generated from the original data. The transformation consists of shears (within 0.3 rad), rotations (within 30 degrees), zooming (within 20% of brain regions), and horizontal reflections, which are randomly created to increase the size of our training dataset by a factor of 1000 through all epochs in both DWI and T2WI images.

### RU-Net for rat brain extraction

**Fig. 1:**
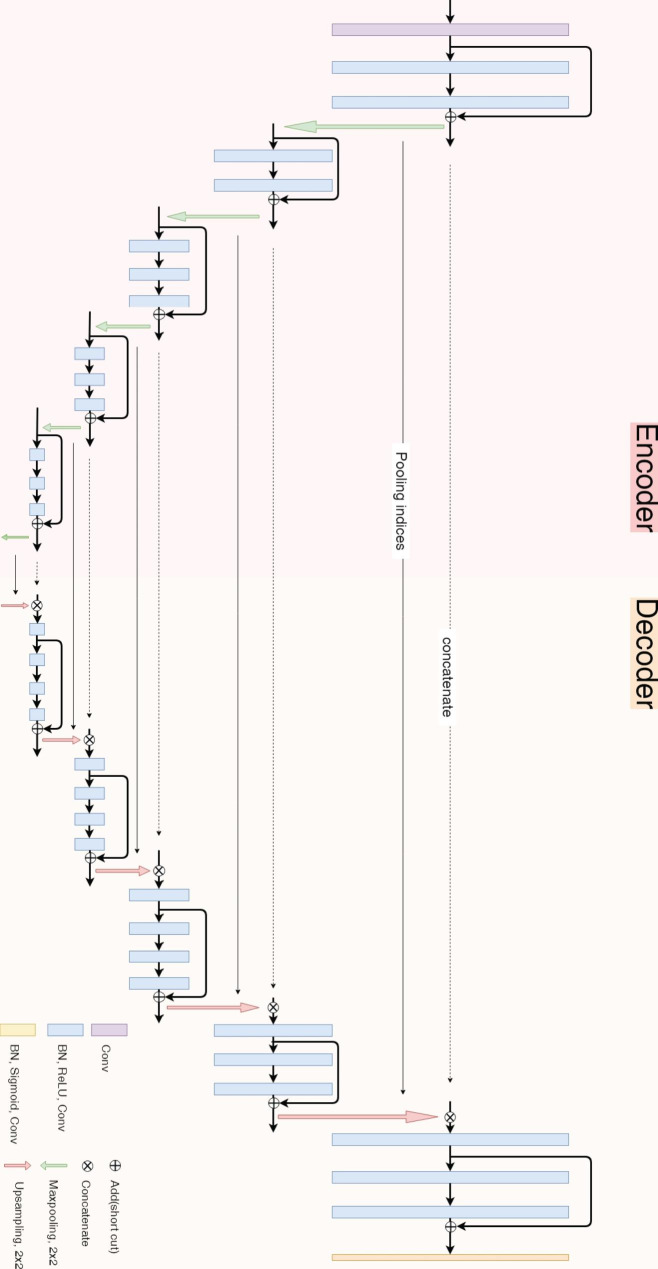
Illustration of the proposed RU-Net architecture

Our RU-Net is a special deep learning framework that takes advantage of the decoupling utility in batch normalization [36], the skip connection in residual network [35], and the feature concatenation in U-Net [28] for skull stripping in pathological rat brain MR images. We introduce the batch normalization and residual network into our encoder-decoder U-Net like architecture to accelerate the convergence speed while reducing the gradient vanishing and explosion problems. As illustrated in Fig. 1, our RU-Net consists of 33 convolutional layers, 5 maximum pooling layers, and 5 upsampling layers. In the encoding path, there are 14 convolutional layers and 5 maximum pooling layers. Each individual rat brain MR image $\widehat{I}$ with a dimension of $N\times N$ is fed into the network in the input layer, followed by a $3\times 3$ convolution process to boost the channel number to 64 in the convolution layer. This $N\times N\times 64$ output provides two functions: input for the subsequent block and input for the residual addition. The block consists of three consecutive layers, namely, batch normalization (BN), activation, and convolution. By normalizing each mini-batch, the BN layer enables us to be less cautious concerning parameter initialization and adopt higher learning rates, which also helps stabilize the network. The rectified linear unit (ReLU) function is utilized in the activation layer followed by a $3\times 3$ convolution layer for feature extraction. The same $N\times N\times 64$ structure is constructed through the entire block, i.e., all three layers. After one additional block with the same architecture, the immediate output and the preserved convolution output are joined together to establish the residual learning network in the addition layer.

Subsequently, the output from the addition layer serves as both the input of the following maximum pooling layer and the concatenation in the decoder phase. The maximum pooling is executed using a $2\times 2$ neighborhood with stride 2 that reduces the output to $\left(N/2\right)\times \left(N/2\right)\times 64$. To tackle large intensity variation in ischemic rat brain images, a pooling index transmission mechanism is introduced so that the corresponding maximum value indices are also stored for recovering the feature locations in the decoding path [40]. The maximum pooling result and its output after two equivalent block processes are united to build a deeper residual learning scheme again in a second addition layer. These encoding procedures of one maximum pooling, three block processing, and one residual addition steps are repeated until the image dimension is scaled down to $\left(N/16\right)\times \left(N/16\right)$. After an additional maximum pooling operation, the decoder phase starts from a contrary $2\times 2$ maximum upsampling layer with stride 2 to produce enlarged features for concatenation. In the deepest concatenation layer, the upsampled result and the output from the deepest addition layer in the encoder phase are integrated into a double channel structure with a dimension of $\left(N/16\right)\times \left(N/16\right)\times 128$. In the following block processing, the output architecture reduces to $\left(N/16\right)\times \left(N/16\right)\times 64$ after the convolution layer. This output and the outcome after three successive blocks are added up to produce the deepest residual learning network in the decoding path. The subsequent maximum upsampling layer again combines the addition result with the output from the corresponding maximum pooling layer in the encoder phase, which expands the outcome to a dimension of $\left(N/8\right)\times \left(N/8\right)\times 64$. This outcome is then concatenated with the output of the matching addition layer in the encoding path to generate a $\left(N/8\right)\times \left(N/8\right)\times 128$ resulting structure.

These procedures associated with upsampling, concatenation, convolution, and residual learning are duplicated until the network dimension grows back to $N\times N\times 64$. In the last block, after the BN layer, the sigmoid function is employed in the activation layer to produce output values between 0 and 1 for segmentation prediction. A final $1\times 1$ convolution layer is utilized to consolidate all channels to a single $N\times N$ probability map, which completes the decoder phase with 19 convolutional layers and 5 upsampling layers. The loss function ${\Lambda }$ is defined in terms of the Dice metric [41] using

2 $${\Lambda }\left({{\Omega }}_{sp},{{\Omega }}_{gt}\right)=1-{\kappa }_{D}\left({{\Omega }}_{sp},{{\Omega }}_{gt}\right)$$

where ${{\Omega }}_{sp}$ represents the segmentation prediction (SP) mask, ${{\Omega }}_{gt}$ represents the ground truth (GT) mask, and ${\kappa }_{D}$ represents the Dice coefficient, which is defined as

3 $${\kappa }_{D}\left({{\Omega }}_{sp},{{\Omega }}_{gt}\right)=\frac{2\left|{{\Omega }}_{sp}\bigcap {{\Omega }}_{gt}\right|}{\left|{{\Omega }}_{sp}\right|+\left|{{\Omega }}_{gt}\right|}=\frac{2{\theta }_{TP}}{{2\theta }_{TP}+{\theta }_{FN}+{\theta }_{FP}}$$

where ${\theta }_{TP}$ represents true positives, ${\theta }_{FN}$ represents false negatives, and ${\theta }_{FP}$ represents false positives associated with ${{\Omega }}_{sp}$ and ${{\Omega }}_{gt}$. To find the best parameters in the proposed RU-Net, the Adam optimizer [42] is employed due to its great effectiveness on computational complexity and memory usage. Varying learning rates with decaying values during the training process are employed to further accelerate the convergence speed.

### Performance evaluation

In addition to the Dice metric as described in Eq. (3), some other evaluation measures are exploited to reveal the correlation between the segmentation and GT masks. Specifically, two similarity metrics of sensitivity ${\kappa }_{st}$ and sensibility ${\kappa }_{sb}$ [43] are adopted to evaluate the degree of under-segmentation and over-segmentation with

4 $${\kappa }_{st}\left({{\Omega }}_{sp},{{\Omega }}_{gt}\right)=\frac{{\theta }_{TP}}{{\theta }_{TP}+{\theta }_{FN}}$$

and

5 $${\kappa }_{sb}\left({{\Omega }}_{sp},{{\Omega }}_{gt}\right)=1-\frac{{\theta }_{FP}}{{\theta }_{TP}+{\theta }_{FN}}$$

, respectively. The Hausdorff distance metric [44], which measures the largest distance of a point set to the nearest point in another, is utilized to signify how close the segmentation and GT contours are in a Euclidean space with

6 $$\begin{array}{l}{\delta _h}\left( {{\Gamma _{sp}},{\Gamma _{gt}}} \right) = \\{\rm{max}}\left( {\mathop {{\rm{max}}}\limits_{s \in {\Gamma _{sp}}} \mathop {{\rm{min}}}\limits_{g \in {\Gamma _{gt}}} \left\| {s - g} \right\|,\mathop {{\rm{max}}}\limits_{g \in {\Gamma _{gt}}} \mathop {{\rm{min}}}\limits_{s \in {\Gamma _{sp}}} \left\| {g - s} \right\|} \right)\end{array}$$

where ${\delta }_{h}$ represents the Hausdorff distance, $\left\| {\, \cdot \,} \right\|$ symbolizes the norm, ${{\Gamma }}_{sp}$ and ${{\Gamma }}_{gt}$ indicate the point sets of the contours corresponding to ${{\Omega }}_{sp}$ and ${{\Omega }}_{gt}$, respectively. A robuster measure of ${\delta }_{h}$ is the average Hausdorff distance that computes the average distance instead of the maximum distance in Eq. (6), which is employed in this study and denoted as ${\delta }_{ah}$. A paired t-test is used to compare the evaluation scores of the proposed framework with those from other methods. A two-tailed P-value < 0.05 is considered statistically significant.

## Results

### Implementation

Our proposed RU-Net framework for rat brain extraction in two different modalities of DWI and T2WI was implemented and programmed in Python 3.5 using Keras 2.1.6 [45]. All experiments were executed on an Intel® Xeon(R) CPU ES-2620 v3 @ 2.40 GHz$\times 24$ workstation running 64-bit Linux Ubuntu 16.04. The machine was equipped with a NVIDIA Tesla K40c GPU of 12GB RAM [46]. The percentages of the training, validation, and testing sets were 6: 2: 2, which were randomly selected from the acquired image datasets. The input image dimensions are $128\times 128$ and $256\times 256$ for DWI and T2WI images, respectively. The training phase was executed using a mini-batch size of 8 with a total number of 100 epochs. The learning rates were initialized with $5{\text{e}}^{-4}$, which gradually decreased to $1{\text{e}}^{-4}$ when the epoch number was larger than 20. The same RU-Net architecture was employed for both DWI and T2WI images but trained individually. There were two different sets of the GT masks corresponding to the DWI and T2WI datasets, which were independently delineated by experienced neurologists in our team. This was mainly because the infarct regions exhibited in DWI and T2WI images were not identical due to different resolution abilities. On the basis of the GT, our skull stripping results were compared with traditional methods including the BSE [47], rBET [48], and RATS [19] as well as the network-based approaches such as the 3-D PCNN [25], DeepMedic [49], and U-Net [32]. For deep-leaning methods of DeepMedic and U-Net, their models were retrained using the same protocols as our RU-Net.

**Fig. 2:**
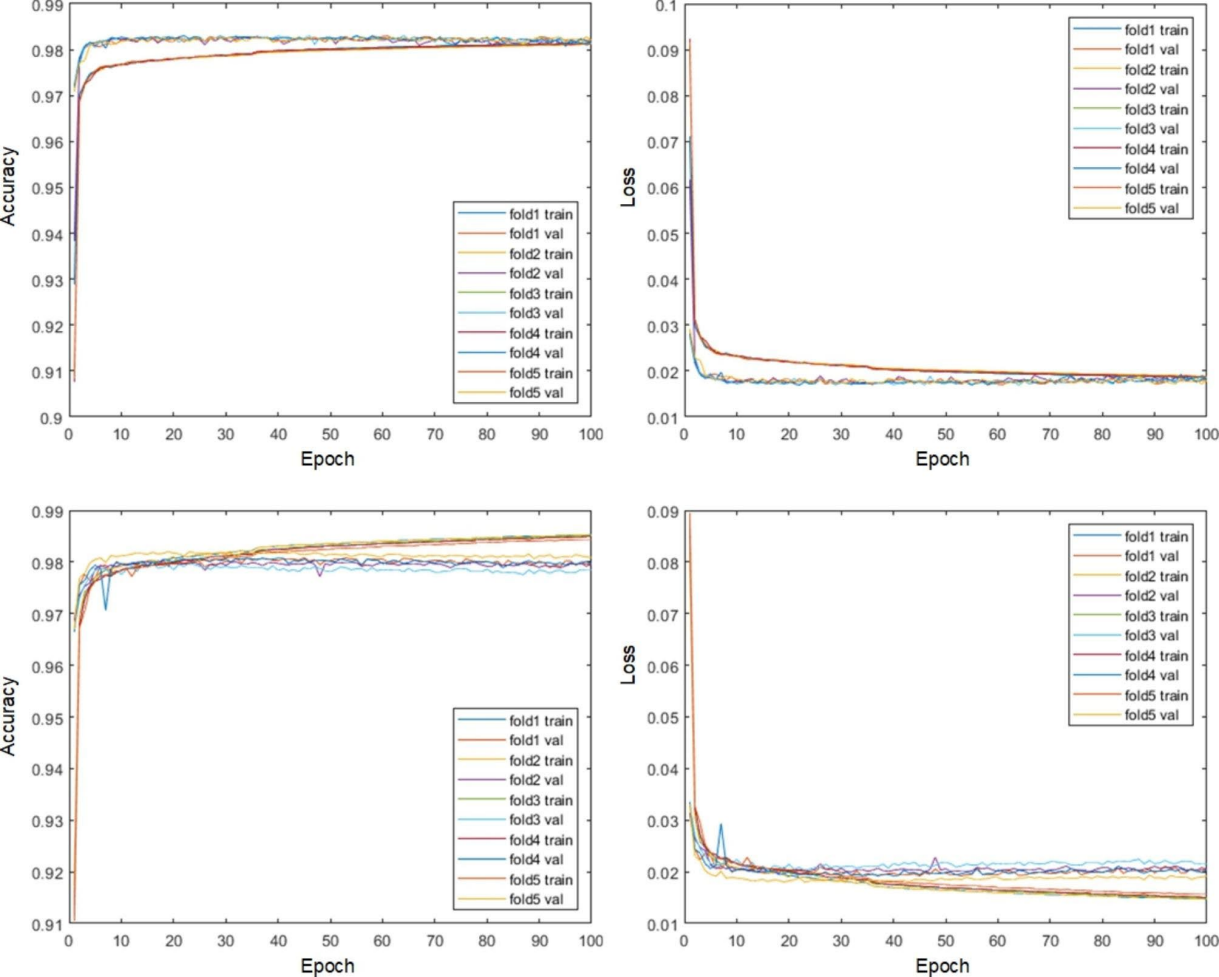
Plots of the accuracy and loss functions using the RU-Net in the training and validation datasets. Top row: DWI subjects. Bottom row: T2WI subjects

### Network cross validation

To understand the effectiveness of the proposed RU-Net skull stripping network, five-fold cross validation was exploited in the training phase. Figure 2 plots the accuracy and loss functions for the training and validation datasets in both DWI and T2WI images. Each fold had two curves that represented the training and validation subjects with respect to the epoch number. All of the five folds exhibited quite similar accuracy and loss trace patterns. For the DWI image scenario, the training curves climbed relatively slowly than the validation curves up towards the same high segmentation accuracy. While the training curves gradually raised their accuracy in T2WI images, the corresponding validation curves reached a plateau and maintained their high accuracy towards the end of the epoch. It was obvious that our RU-Net achieved high skull stripping accuracy with tiny loss in the DWI and T2WI rat brain image datasets, which indicated the robustness of our developed network. Further validation on the RU-Net segmentation performance was presented in Table 1 in comparison with different architecture variants using $3\times 3$ and $4\times 4$ maximum pooling, $7\times 7$ convolution, and 4 level U-shape network structure. It was apparent that the proposed RU-Net architecture exhibited the best evaluation scores with the narrowest standard deviations in terms of ${\kappa }_{D}$, ${\kappa }_{st}$, and ${\kappa }_{sb}$.

**Table 1:** Segmentation performance comparison between different network architecture settings

Network architecture	Evaluation metric		
	Κ_D_(%)	Κ_st_(%)	Κ_sb_(%)
Maximum pooling:$3\times 3$	95.20 ± 2.53	96.32 ± 3.23	93.77 ± 4.36
Maximum pooling:$4\times 4$	95.18 ± 2.61	96.24 ± 3.29	93.82 ± 4.55
Convolution filter:$7\times 7$	95.96 ± 2.42	96.92 ± 2.97	94.88 ± 2.98
U-shape structure depth: 4	98.01 ± 1.50	97.78 ± 1.85	98.06 ± 1.87
RU-Net	**98.04 ± 0.33**	**97.94 ± 0.75**	**98.15 ± 0.68**

### DWI skull stripping

**Fig. 3:**
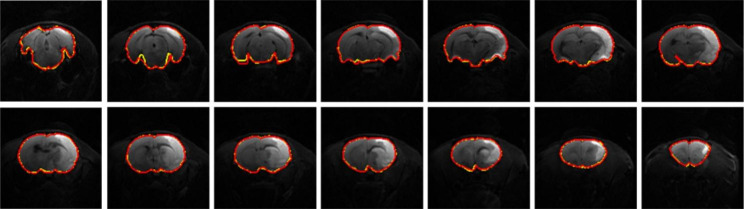
Illustration of DWI (Subject 39) skull stripping results using the proposed RU-Net framework. Yellow: Prediction. Red: GT

**Fig. 4:**
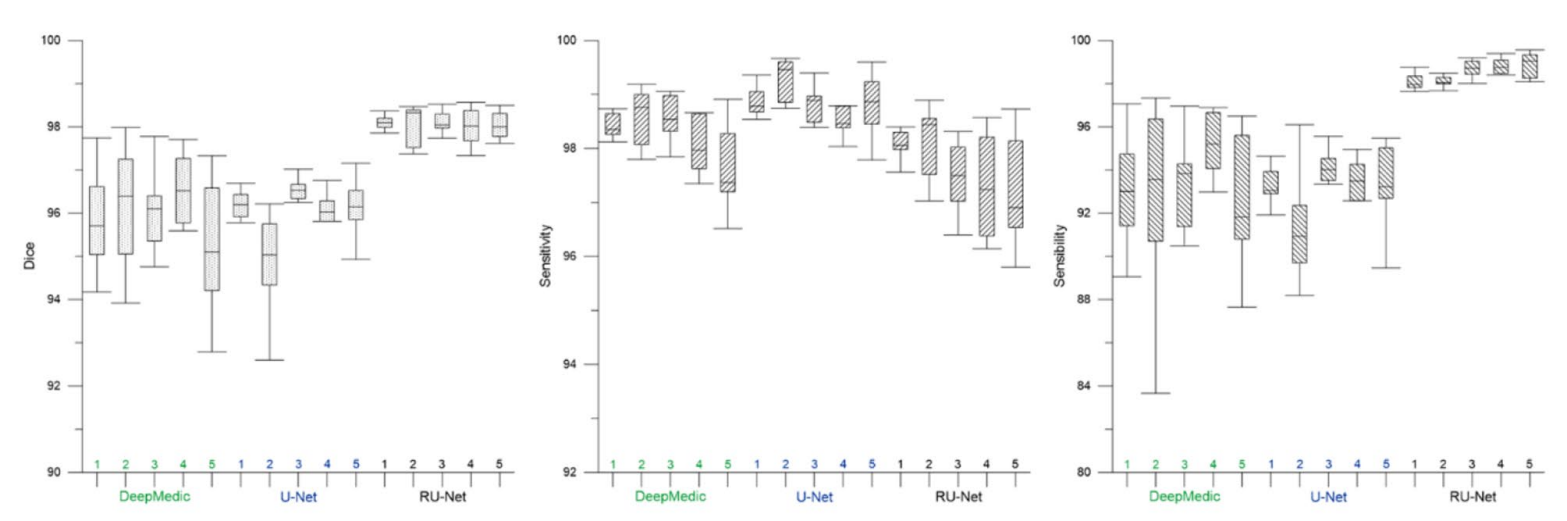
Performance analyses of DWI skull stripping results based on five-fold cross validation

**Fig. 5:**
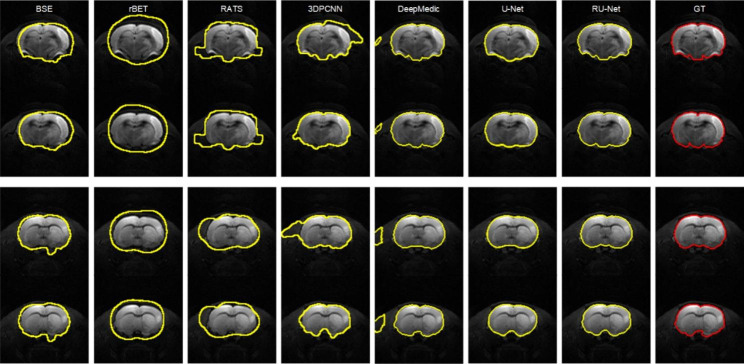
Visual comparison of DWI skull stripping results using different methods. Top row: slices 7 and 8 of Subject 10. Bottom row: slices 9 and 10 of Subject 21

**Fig. 6:**
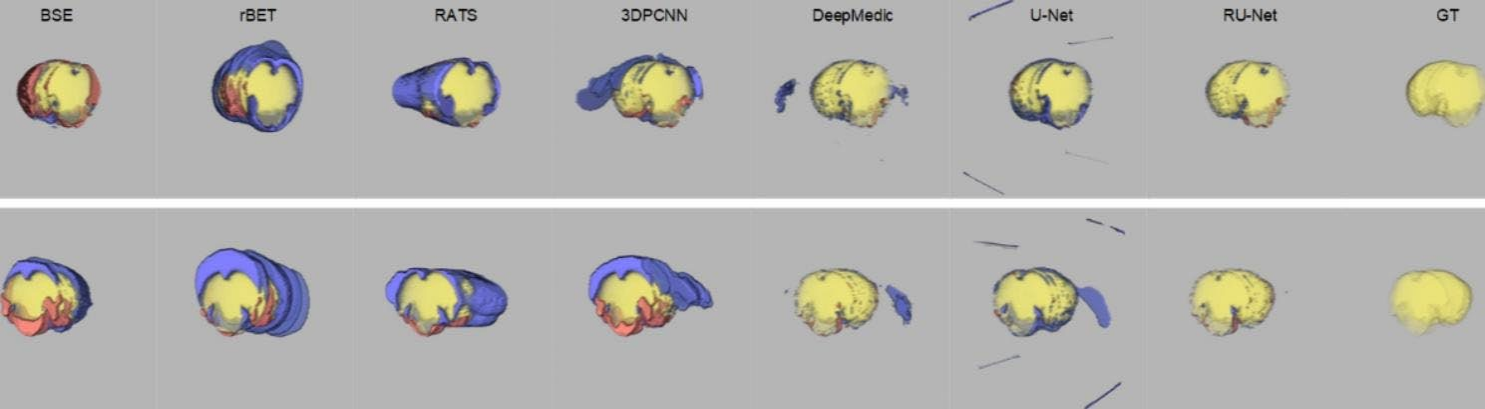
Visual comparison of DWI skull stripping results in 3-D view using different methods. Blue: ${\theta }_{FP}$. Red: ${\theta }_{FN}$. Top row: Subject 16. Bottom row: Subject 40

Figure 3 illustrates qualitative skull stripping results in a sequence of DWI images using the proposed scheme along with the corresponding GT masks. The segmented brain regions (yellow) were observed to be well conformed to the GT contours (red). Performance measures of the skull stripping results using the Dice, sensitivity, and sensibility metrics based on five-fold cross validation were depicted in Fig. 4. It was noted that the proposed RU-Net produced the highest average Dice and sensibility scores with the narrowest standard deviations over the DeepMedic and U-Net methods. While the average sensitivity scores of the three methods were somewhat overlapped, the U-Net was slightly higher than other two methods. Representative skull stripping results using the abovementioned seven methods were qualitatively illustrated in Fig. 5. All approaches more or less encompassed the rat brain regions but the BSE, rBET, RATS, 3-D PCNN, and DeepMedic methods revealed apparent false positive regions. Both U-Net and RU-Net produced accurate segmentation results with the U-Net contours more smooth and the RU-Net contours deformed into the fissures, which better resembles the GT. Figure 6 demonstrates visual skull stripping results of two different subjects with DWI in 3-D view. Obvious over-segmentation and under-segmentation outcomes were generated by the traditional methods of BSE, rBET, RATS, and 3-D PCNN. More precise results were obtained using the deep learning-based methods. While the segmentation masks provided by the DeepMedic and U-Net methods were with excess components, our RU-Net scheme generated clean rat brain regions. Table 2 summarizes statistical analyses of the skull stripping results in the DWI image dataset in terms of the four evaluation metrics. The proposed RU-Net framework achieved the highest average evaluation scores of ${\kappa }_{D}=98.04\%$ ($p<0.001$) and ${\kappa }_{sb}=98.15\%$ ($p<0.001$) with the ${\kappa }_{st}$ score slightly smaller than the maximum value received by the U-Net method. Our segmentation performance was further validated by the smallest average value of ${\delta }_{ah}=0.1161$mm ($p<0.001$) compared with all competitive methods.

**Table 2:** Quantitative comparison of rat skull stripping results in DWI image volumes between different methods

Method	Evaluation metric			
	Κ_D_(%)	Κ_st_(%)	Κ_sb_(%)	δ_ah_(mm)
BSE	81.71 ± 2.43	72.49 ± 2.31	89.97 ± 2.34	2.4219 ± 0.8785
rBET	82.69 ± 2.76	97.21 ± 2.91	63.28 ± 7.82	2.2802 ± 0.6547
RATS	86.12 ± 3.51	91.56 ± 2.87	77.89 ± 3.86	1.7673 ± 0.7891
3DPCNN	88.87 ± 5.24	96.74 ± 1.53	78.57 ± 3.31	1.6485 ± 0.8637
DeepMedic	95.88 ± 1.43	98.24 ± 0.62	93.27 ± 3.05	0.9896 ± 0.7955
U-Net	95.87 ± 0.97	**98.76 ± 1.00**	92.71 ± 2.43	0.9298 ± 0.6795
RU-Net	**98.04 ± 0.33**	97.94 ± 0.75	**98.15 ± 0.68**	**0.1161 ± 0.0754**

**Fig. 7:**
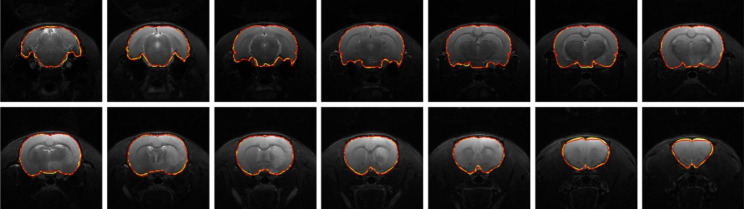
Illustration of T2WI (Subject 31) skull stripping results using the proposed RU-Net framework. Yellow: Prediction. Red: GT

**Fig. 8:**
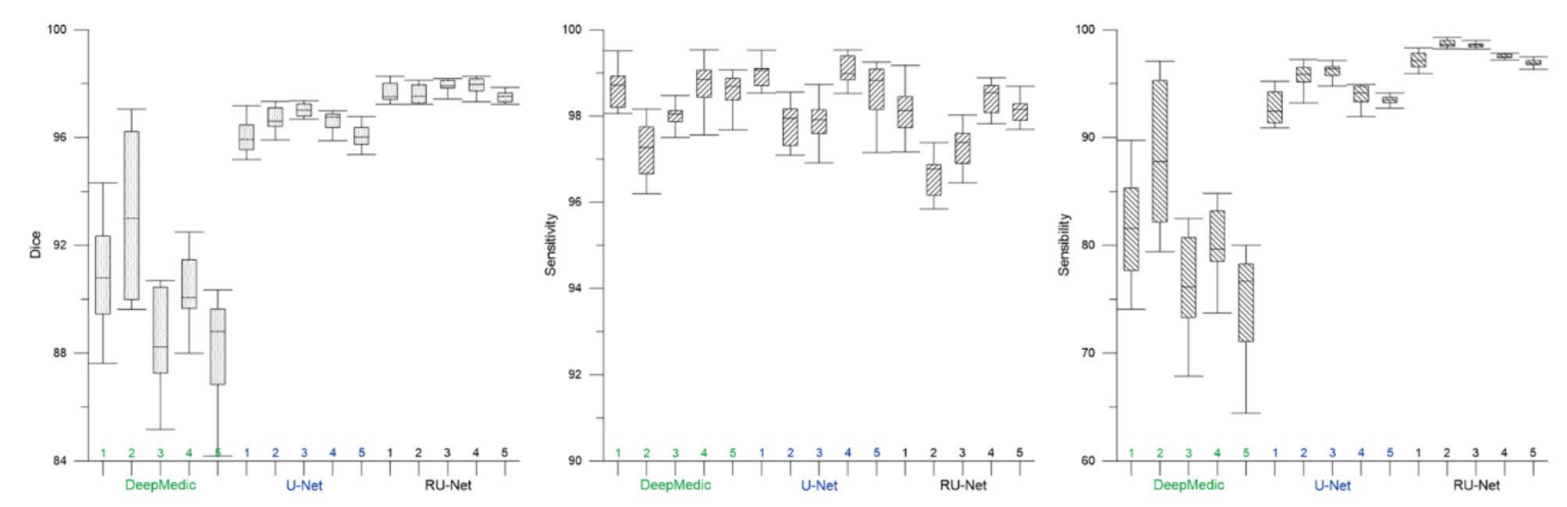
Performance analyses of T2WI skull stripping results based on five-fold cross validation

### T2WI skull stripping

**Fig. 9:**
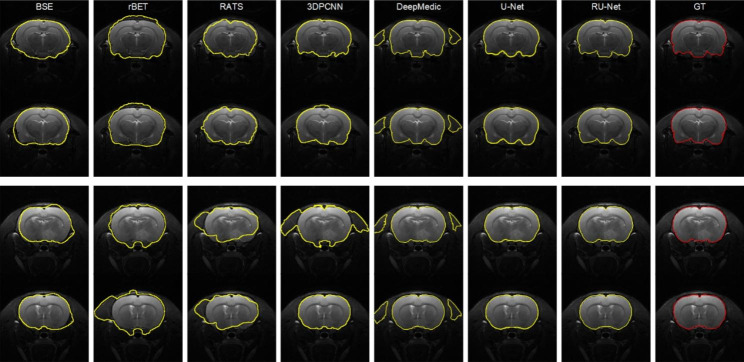
Visual comparison of T2WI skull stripping results using different methods. Top row: slices 6 and 7 of Subject 9. Bottom row: slices 8 and 9 of Subject 33

**Fig. 10:**
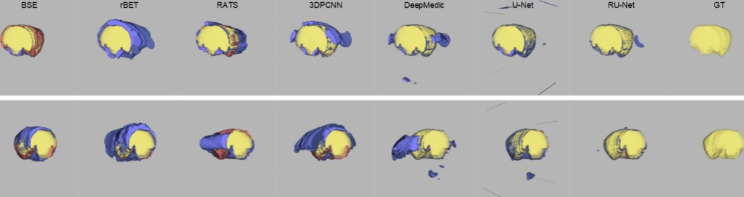
Visual comparison of T2WI skull stripping results in 3-D view using different methods. Blue: ${\theta }_{FP}$. Red: ${\theta }_{FN}$. Top row: Subject 3. Bottom row: Subject 37

In the scenario of T2WI image segmentation, the proposed RU-Net scheme also performed well. As illustrated in Fig. 7, the segmented brain regions (yellow) were decently similar to the corresponding GT masks (red) in all instances. Figure 8 shows quantitative evaluation of the skull stripping results in the T2WI dataset based on five-fold cross validation. The average Dice and sensibility scores provided by our RU-Net architecture were higher and with smaller standard deviations than the DeepMedic and U-Net methods. The overlapping phenomena of the average sensitivity scores between the three methods in the T2WI subjects were more evident than the DWI subjects. We visually compared our skull stripping framework with the seven methods in Fig. 9, where two randomly selected subjects were presented. Similar to the DWI segmentation scenario, there were noticeable false positive regions in some slices using the BSE, rBET, RATS, 3-D PCNN, and DeepMedic methods. The U-Net generated smooth contours that approximately circumscribed the rat brain surfaces, whereas the proposed RU-Net achieved more accurate contours that were better compatible with the GT. Figure 10 compares the whole skull stripping outcomes of Subjects 3 and 37 in 3-D view between different methods. Apparent segmentation errors were observed using the BSE, rBET, RATS, 3-D PCNN, and DeepMedic methods. Both U-Net and RU-Net schemes produced more precise segmentation results with fewer flaws. Nevertheless, our RU-Net achieved higher Dice scores of 97.25% and 98.08% for Subjects 3 and 37, respectively. Statistical analyses of the rat brain segmentation results in T2WI image volumes in Table 3 indicated our advantage over other methods with the highest average values of ${\kappa }_{D}=97.67\%$ ($p<0.001$) and ${\kappa }_{sb}=97.42\%$ ($p<0.001$). Lastly, the smallest average score of ${\delta }_{ah}=0.1406$mm ($p<0.001$) attained by the RU-Net further confirmed our skull stripping efficacy.

**Table 3:** Quantitative comparison of rat skull stripping results in T2WI image volumes between different methods

Method	Evaluation metric			
	Κ_D_(%)	Κ_st_(%)	Κ_sb_(%)	δ_ah_(mm)
BSE	82.44 ± 2.40	73.13 ± 2.27	89.99 ± 2.31	2.3127 ± 0.8563
rBET	83.51 ± 3.56	**98.93 ± 0.26**	65.04 ± 8.63	2.0538 ± 0.6435
RATS	87.31 ± 4.12	93.16 ± 2.77	78.97 ± 7.68	1.6806 ± 0.7611
3DPCNN	89.06 ± 4.18	97.25 ± 2.81	78.69 ± 8.06	1.6132 ± 0.8527
DeepMedic	90.20 ± 2.74	98.23 ± 0.74	80.20 ± 6.90	1.5440 ± 0.6359
U-Net	96.43 ± 0.65	98.35 ± 1.17	94.38 ± 1.66	0.6850 ± 0.4451
RU-Net	**97.67 ± 0.46**	97.90 ± 1.00	**97.42 ± 0.97**	**0.1406 ± 0.1357**

## Discussion

A new skull stripping framework for pathological rat brain MR images in light of deep learning networks has been introduced. The development of this RU-Net was inspired by the demand for preclinical stroke investigation associated with both DWI and T2WI image volumes. As the U-Net [28] has been successfully employed in many medical image segmentation applications [28, 32, 33], our network took advantage of the U-shape architecture from the U-Net. To handle the nonuniform intensity distribution and blurred brain boundaries in the ischemic rat MR images, a series of BN layers conceived from the batch normalization scheme [36] constituted the block structure in the encoding and decoding paths. Enhancement learning was accomplished by a residual network [35] that connects the input with the output features of each block in both encoder and decoder. A common disadvantage of deep learning-based approaches for medical image processing is the limited number of image data comparing to the scale of natural image databases such as the ImageNet. We tackled this issue by augmenting existing image data through different spatial transformations to diversify the training data. Based on the five-fold cross validation with the rat brain MR image datasets, we updated and finalized the system parameters to achieve the optimal architectures. Different evaluation metrics associated with the paired t-test were employed to compare our segmentation outcome with the state-of-the-art methods.

As presented in Tables 2 and 3, comparable skull stripping results were obtained in both the DWI and T2WI image datasets using the traditional methods of BSE, rBET, RATS, and 3-D PCNN. Developed for human brain image segmentation, the BSE method produced acceptable skull stripping results around the middle slices of the rat brain image volumes. However, notable segmentation errors appeared roughly in the first and last three slices, which deteriorated the overall performance. Modified from the BET scheme, the rBET method also adopted an active contour model that was evaluated in rat brain T1-weighted and T2-weighted MR images. Obvious over-segmentation outside the rat brain boundaries reduced its segmentation accuracy due to the abnormity in the DWI and T2WI image datasets, leading to the poorest sensibility scores in both scenarios. Originally validated on normal rat brain MR images similar to the rBET method, the RATS algorithm was unable to efficiently separate the ischemic rat brain regions from the surrounding tissues, particularly for DWI images. Extended from the 2-D PCNN model and verified in mouse brain T2WI images, the 3-D PCNN algorithm generated unstable skull stripping results so that some slices exhibited apparent false positive regions in the ischemic rat image datasets as illustrated in Figs. 5 and 9.

**Fig. 11:**
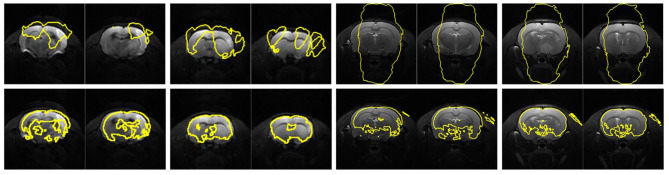
Visual skull stripping results using the original MU-Net (top row) and MedicDeepLabv3+ (bottom row) without retraining. Left columns: DWI Subjects 10 and 21. Right columns: T2WI Subjects 9 and 33

Different from the traditional approaches, the deep learning-based models exploited an end-to-end network, which usually provide better outcomes. As can be realized from the evaluation scores, the DeepMedic, U-Net, and RU-Net schemes exhibited higher skull stripping accuracy with smaller ${\delta }_{ah}$ values in both DWI and T2WI scenarios. Equipped with the efficient multiscale 3-D CNN and fully connected conditional random field model, the DeepMedic scheme adequately captured the rat brain surfaces. Likewise, the skull stripping results using the U-Net method decently enclosed the rat brains in all demonstrated instances. Due to the large ${\theta }_{FP}$ regions, the U-Net exhibited low ${\kappa }_{sb}$ scores, which in turn produced higher ${\kappa }_{st}$ scores than our RU-Net. To segment the rat brain MR images with ischemia, both DeepMedic and U-Net models were retrained using the same protocols as our RU-Net to fine-tune their system parameters. All three deep learning-based frameworks were evaluated according to the five-fold cross validation in the DWI and T2WI image datasets as revealed in Figs. 4 and 8, respectively. Statistical analyses using the Dice, sensitivity, and sensibility metrics indicated convergent characteristics of the three networks. This was mainly because the deep learning mechanisms were refreshed to adapt the systems to new image data. Without the retraining process for parameter adjustments, the segmentation to unfamiliar image data could be improper. To illustrate this, Fig. 11 depicts the skull stripping results of the same slices and subjects in Figs. 5 and 9 using the original models of the MU-Net [33], which was originally developed for large mouse brain segmentation in T2WI images, and the MedicDeepLabv3+ [34]. Their average ${\kappa }_{D}$ scores were 31.01% and 34.72% for the DWI dataset, and 55.46% and 48.28% for the T2WI dataset, respectively.

One inevitable shortage of deep learning-based strategies for medical image segmentation is that the outcome may exhibit disconnected components with broken pieces and interior holes. This is mainly due to the natural characteristics of pixel-to-pixel partition based on the feature maps at different scales and depths. Although the consecutive convolution processes include neighboring information, the involvement is too shallow and limited mostly to adjacent pixels. For natural images, this partition scheme will not cause serious issues as the color information of three channels is involved and the intensity variation is relatively subtle. For medical images as in our scenario, the single gray scale image is the only input to the system and inhomogeneous intensities are obviously presented. As shown in Figs. 6 and 10, noticeable false positive regions apart from the brains were produced using the DeepMedic and U-Net methods. Thanks to the unique network architecture, the proposed RU-Net faithfully delineated the rat brain boundaries and achieved accurate skull stripping results with minor over-segmentation errors compared to other networks. This is not only because our architecture contains the BN layer associated with the residual network but also because the salient feature locations in the encoder are transmitted to the corresponding upsampling procedures in the decoder to strengthen the spatial correlation. From the perspective of practical applications, the outcome from deep learning-based approaches can be improved by appropriate morphological operations to acquire clean and complete brains. For example, the average sensibility scores of the DeepMedic and U-Net schemes in the DWI image dataset increased to 97.50% and 93.11%, respectively, and they advanced to 97.14% and 94.59% in the T2WI image dataset. Lastly, our RU-Net can be extended for multimodal learning by feeding, say, two different modalities of DWI and T2WI images to the corresponding network and integrating the intermediate results through an extra concatenation structure to generate the ultimate prediction.

## Conclusion

In this paper, we investigated an automatic skull stripping framework in pathological rat brain MR images in light of a deep learning architecture, namely RU-Net. Motivated by the demand of segmenting rat brain MR images after ischemic stroke, the proposed scheme was established on an efficient U-shape like network with embedded BN layers reinforced by the residual network. A variety of ischemic rat brain images in two different DWI and T2WI datasets were employed to evaluate the capability of our rat brain segmentation network. Comparable performance with high evaluation scores in terms of Dice, sensitivity, and sensibility was observed in both image datasets. Our RU-Net outperformed the state-of-the-art methods either traditional mathematics models or deep learning networks in extracting clean rat brain regions with a nonuniform intensity distribution in the acquired MR image volumes. We believe that the proposed skull stripping network is of great potential for advancing preclinical stroke investigation as well as providing an efficient tool for abnormal rat brain MR image extraction, where accurate segmentation of the brain region is fundamental.

## Data Availability

The data that support the findings of this study are available for sharing from the corresponding authors upon reasonable request.

## Abbreviations

BET: brain extraction tool
BN: batch normalization
CCA: common carotid artery
CNN: convolutional neural network
DWI: diffusion weighted imaging
FCN: fully convolutional network
FOV: field of view
GT: ground truth
ICA: internal carotid artery
MRI: magnetic resonance imaging
MU-Net: multi-task U-Net
PCNN: pulse coupled neural network
RBD: rat brain deformation
ReLU: rectified linear unit
RU-Net: Rat U-Net
SHERM: SHape descriptor selected Extremal Regions after Morphologically filtering
SP: segmentation prediction
T2WI: T2-weighted MRI
TE: echo time
tMCAO: transient middle cerebral artery occlusion
TR: repetition time
